# Supplementary material for: β-catenin inhibition disrupts the homeostasis of osteogenic/adipogenic differentiation leading to the development of glucocorticoid-induced osteonecrosis of the femoral head
Source: eLife. 2024 Feb 20;12:RP92469. doi: 10.7554/eLife.92469 (PMC10942600; doi:10.7554/eLife.92469)
Supplement: Supplementary file 1. [file elife-92469-supp1.docx]

Supplementary File 1. The information of patients provided femoral head samples

| GONFH | Age | Sex | Admission number | Hospital |
| --- | --- | --- | --- | --- |
| Case 1 | 40 | M | 01092374 | The First Affiliated Hospital of Zhejiang Chinese Medical University |
| Case 2 | 58 | F | 00446464 | The First Affiliated Hospital of Zhejiang Chinese Medical University |
| Case 3 | 39 | F | 00465298 | The First Affiliated Hospital of Zhejiang Chinese Medical University |
| Case 4 | 51 | M | 00470866 | The First Affiliated Hospital of Zhejiang Chinese Medical University |
| Case 5 | 56 | F | 01152074 | The First Affiliated Hospital of Zhejiang Chinese Medical University |
| Case 6 | 37 | M | 01161839 | The First Affiliated Hospital of Zhejiang Chinese Medical University |
| Case 7 | 54 | M | 01168786 | The First Affiliated Hospital of Zhejiang Chinese Medical University |
| Case 8 | 64 | M | 01187289 | The First Affiliated Hospital of Zhejiang Chinese Medical University |
| Case 9 | 58 | M | 01168321 | The First Affiliated Hospital of Zhejiang Chinese Medical University |
| Case 10 | 34 | M | 01181984 | The First Affiliated Hospital of Zhejiang Chinese Medical University |
| Case 11 | 57 | M | 01140305 | The First Affiliated Hospital of Zhejiang Chinese Medical University |
| Case 12 | 34 | M | 01182095 | The First Affiliated Hospital of Zhejiang Chinese Medical University |
| Case 13 | 45 | M | 01184637 | The First Affiliated Hospital of Zhejiang Chinese Medical University |
| Case 14 | 58 | F | 01187399 | The First Affiliated Hospital of Zhejiang Chinese Medical University |
| Case 15 | 35 | F | 01192770 | The First Affiliated Hospital of Zhejiang Chinese Medical University |
| Femoral head fracture |  |  |  |  |
| Case 1 | 70 | F | 8243003 | Jiangnan Hospital Affiliated to Zhejiang Chinese Medical University |
| Case 2 | 76 | M | 81632863 | Jiangnan Hospital Affiliated to Zhejiang Chinese Medical University |
| Case 3 | 90 | F | 01056750 | The First Affiliated Hospital of Zhejiang Chinese Medical University |
| Case 4 | 80 | F | 01056747 | The First Affiliated Hospital of Zhejiang Chinese Medical University |
| Case 5 | 69 | M | 1089539 | The First Affiliated Hospital of Zhejiang Chinese Medical University |
| Case 6 | 78 | M | 157468 | The Second Affiliated Hospital of Zhejiang Chinese Medical University |
| Case 7 | 84 | M | 156570 | The Second Affiliated Hospital of Zhejiang Chinese Medical University |
| Case 8 | 72 | M | 157453 | The Second Affiliated Hospital of Zhejiang Chinese Medical University |
| Case 9 | 91 | M | 80736687 | Jiangnan Hospital Affiliated to Zhejiang Chinese Medical University |
| Case 10 | 73 | M | 70688222 | Jiangnan Hospital Affiliated to Zhejiang Chinese Medical University |
