# Supplementary material for: β-catenin inhibition disrupts the homeostasis of osteogenic/adipogenic differentiation leading to the development of glucocorticoid-induced osteonecrosis of the femoral head
Source: eLife. 2024 Feb 20;12:RP92469. doi: 10.7554/eLife.92469 (PMC10942600; doi:10.7554/eLife.92469)
Supplement: Supplementary file 2. [file elife-92469-supp2.docx]

Supplementary File 2. The primer sequences of mouse genotype

| Mouse Genotype | Primer sequence | |
| --- | --- | --- |
| *Col2a1-CreER*  Mut (200 bt) | Forward | 5'-ATTGCTGTCACTTGGTCGTGGC-3' |
|  | Reverse | 5'-GAAAATGCTTCTGTCCGTTTGC-3' |
| *Sp7-CreER*  Mut (420 bp) | Forward | 5'-CTGGTCTGGACACAGTGCCCG-3' |
|  | Reverse | 5'-TGCCAGGTTGGTCAGTAAGCC-3' |
| *Rosa26-LSL-tdTomato*  Mut (196 bp) | Forward | 5'-GGCATTAAAGCAGCGTATCC-3' |
|  | Reverse | 5'-CTGTTCCTGTACGGCATGG-3' |
| *Rosa26-LSL-tdTomato*  Wt (297 bp) | Forward | 5'-AAGGGAGCTGCAGTGGAGTA-3' |
|  | Reverse | 5'-CCGAAAATCTGTGGGAAGTC-3' |
| *Ctnnb1*^flox/flox^  Mut (324 bp) | Forward | 5'-AAGGTAGAGTGATGAAAGTTGTT-3' |
|  | Reverse | 5'-CACCATGTCCTCTGTCTATTC-3' |
